# COVID-19 in kidney transplantation-implications for immunosuppression and vaccination

**DOI:** 10.3389/fmed.2022.1060265

**Published:** 2022-11-23

**Authors:** Lavanya Kodali, Pooja Budhiraja, Juan Gea-Banacloche

**Affiliations:** ^1^Department of Internal Medicine, Mayo Clinic, Phoenix, AZ, United States; ^2^Division of Nephrology, Transplant Center, Mayo Clinic, Phoenix, AZ, United States; ^3^Division of Clinical Research, National Institute of Allergy and Infectious Diseases, Bethesda, MD, United States

**Keywords:** COVID-19 vaccine response, KTR, heterologous vaccination, immunosuppression, monoclonal antibodies, solid organ transplant recipients

## Abstract

COVID-19 pandemic continues to challenge the transplant community, given increased morbidity and mortality associated with the disease and poor response to prevention measures such as vaccination. Transplant recipients have a diminished response to both mRNA and vector-based vaccines compared to dialysis and the general population. The currently available assays to measure response to vaccination includes commercially available antibody assays for anti-Spike Ab, or anti- Receptor Binding Domain Ab. Positive antibody testing on the assays does not always correlate with neutralizing antibodies unless the antibody levels are high. Vaccinations help with boosting polyfunctional CD4+ T cell response, which continues to improve with subsequent booster doses. Ongoing efforts to improve vaccine response by using additional booster doses and heterologous vaccine combinations are underway. There is improved antibody response in moderate responders; however, the ones with poor response to initial vaccination doses, continue to have a poor response to sequential boosters. Factors associated with poor vaccine response include diabetes, older age, specific immunosuppressants such as belatacept, and high dose mycophenolate. In poor responders, a decrease in immunosuppression can increase response to vaccination. COVID infection or vaccination has not been associated with an increased risk of rejection. Pre- and Post-exposure monoclonal antibodies are available to provide further protection against COVID infection, especially in poor vaccine responders. However, the efficacy is challenged by the emergence of new viral strains. A recently approved bivalent vaccine offers better protection against the Omicron variant.

## Introduction

COVID-19 pandemic has been a major challenge for the transplant community with associated increased hospitalizations, need for mechanical ventilation, acute kidney injury, kidney graft loss and mortality up to 25% (1). There have been increased efforts to augment immunity with vaccination, provide monoclonal antibodies for prophylaxis and treatment, and antiviral medications. Here we review these topics in detail particularly with respect to kidney transplant recipients (KTRs), but also other solid organ transplant recipients in general.

Listed below is a brief overview of the topics discussed in this review:

1. Measurement of immune response after COVID-19 vaccination.
  a) Overview of humoral response, neutralizing antibodies and cellular response.
  b) Type of vaccines currently available and their mechanism of action.
  c) Measurement of humoral and neutralizing antibody response after vaccination.
  d) Measurement of cellular immunity after vaccination.
2. Response rates to COVID 19 Vaccination in kidney and other solid organ transplant recipients.
3. Factors associated with poor response to vaccination.
4. Response to multiple doses of vaccination.
5. Heterologous Vaccination Strategy.
6. Vaccination response with decreased immunosuppression.
7. Pre and Post exposure Prophylaxis.

Other immunosuppression considerations.

## Measurement of immune response after COVID-19 vaccination

Infection with SARS-CoV-2 results in a strong humoral and cellular immune response. Antibodies (IgG, IgA and IgM) are typically detected between 5 and 15 days after symptom onset, with IgM appearing first and IgG being the most durable. Levels of antibody are higher and persist longer in more severe disease (2). Among the coronavirus structural proteins, the Spike (S) and the Nucleocapsid (N) proteins are the main immunogens (3). The S protein consists of two subunits, S1 which contains the Receptor Binding Domain (RBD) and S2.

### Humoral response, neutralizing antibodies and cellular response

The various antibody assays include antibodies to the S1 and/or S2, the RBD or the N protein. Measurements of antibodies can test their functional ability to neutralize the virus (neutralizing antibodies, costly and time consuming) or just the binding to antigenic targets (binding antibodies, easier to measure and widely available). High level of binding antibodies correlates with neutralizing activity. Neutralizing antibodies correlate with protection against COVID-19 disease, and binding antibodies correlate with neutralizing antibodies. Consequently, what is available to clinicians involved in the care of transplant recipients is a surrogate of a surrogate, and hence far from perfect.

Cellular immune responses against the virus, although considered essential to control the infection, are not routinely measured.

Immunization, as opposed to natural infection, is fundamentally constrained to generate an immune response only against the portions of SARS-CoV-2 included in the vaccine. Most available vaccines include only a fragment of the spike protein (either as a protein or as the RNA or DNA template that will generate it *in vivo*) and so evokes antibody to S, particularly the Receptor Binding Domain on the S1 subunit of the spike protein (4). The RBD interacts with ACE2 (Angiotensin-Converting Enzyme 2) receptor present on the epithelial cells of the bronchi and pulmonary alveoli, arteries, heart, kidneys and brain. The RBD or Spike Ab prevent the virus from penetrating the cells by inhibiting its binding to the ACE2 receptor. Vaccination does not elicit antibodies to Nucleocapsid, so the presence of anti-N IgG or IgM may identify individuals who had recent or prior COVID-19 infection. Antibodies to anti-S persist longer than anti-N (5).

### Type of vaccines currently available and their mechanism of action

Several vaccines are available worldwide. Based on the technology used, these vaccines can be classified as whole virus, protein subunit, nucleic acid or viral vector. As of this writing, the World Health Organization (WHO) includes the following vaccines in their Emergency Use Listing (EUL) (Table 1).

**Table 1:** Types of COVID 19 vaccines.

**Vaccines currently approved by WHO for emergency use listing (EUL)**	**Type of vaccine**
Pfizer/BioNTech Comirnaty vaccine (BNT162b2)	Nucleic acid, mRNA
Spikevac Moderna COVID-19 vaccine (mRNA 1,273)	Nucleic acid, mRNA
Convidecia CanSinoBIO Ad5-nCoV-S vaccine	Viral vector
Vaxzevria/Covishield vaccine/AstraZeneca (ChAdOx1-S)	Viral vector
Janssen/Ad26.COV 2.S vaccine	Viral vector
Sinopharm COVID-19 vaccine	Whole inactivated virus
Sinovac-CoronaVac vaccine	Whole inactivated virus
Bharat Biotech BBV152 Covaxin vaccine	Whole inactivated virus
Covovax vaccine (NVX-CoV2373)	Protein subunit, whole recombinant spike
Nuvaxovid by Novavax (NVX-CoV2373)	Protein subunit, whole recombinant spike
Bivalent Vaccine (Original and Omicron BA.4/BA.5) Pfizer or Moderna^*^	Nucleic acid, mRNA

* Not WHO approved, but FDA approved.

The mRNA vaccines contain genetically engineered mRNA which encodes a fragment of the S protein. In the vector vaccine, a replication-incompetent adenovirus carrying the DNA sequence that encodes a fragment of Spike is used. The adenovirus enters human cells, and these produce the fragment of the spike protein, which elicits an immune response, including antibody production (anti-S) protein. Vector vaccines have been associated with thrombotic complications and are not commonly used in the US or Canada. The Nuvaxovid vaccine includes the whole spike protein. Although there is data on its efficacy and immunogenicity in the general population, there is paucity of such data in kidney transplant recipients (6, 7).

### Measurement of humoral and neutralizing antibody response after vaccination

Humoral response to vaccination can be measured by using anti-S or anti-RBD immunoglobulin G (IgG) level and by the percentage of neutralizing antibody inhibition measured with a surrogate viral neutralization test (%SVNT). Measurement of neutralizing antibodies is costly and time consuming but measuring antibody levels by enzyme linked immunoassay (ELISA) is widely available. Importantly, high titers of anti-spike IgG which can be measured by commercial serological tests correlate well with the presence of neutralizing antibodies and vaccine efficacy against primary symptomatic COVID-19 in the general population. Correlation is better with prevention of symptomatic disease than with prevention of asymptomatic infection.

It is important to understand there are dozens of FDA-authorized antibody tests, and that the levels measured by each test may not be comparable. Depending on the test, a result may be given in optical density (OD), arbitrary units (AU), binding antibody units (BAU) or some other proprietary measurement. There is a standard developed by the World Health Organization (WHO), but WHO international units (IU) are not routinely reported.

A study reported that anti-S1 IgG Ab level of 264 binding antibody units (BAU)/ml and anti RBD Ab of 506 BAU/ml were associated with 80% vaccine efficacy against primary symptomatic COVID-19 infection in the general population. Values of 26 IU/ml for pseudo virus neutralizing antibody titers, and 247 IU/mL for live virus neutralizing antibody titers were also shown to be associated with 80% vaccine efficacy against symptomatic infection (8). No protective “threshold titer” was identified.

In the corona virus efficacy (COVE) phase 3 trial of mRNA 1,273 vaccine, all antibody markers were inversely associated with COVID 19 risk. Vaccine recipients with postvaccination 50% neutralization titers 10, 100, and 1,000 had estimated vaccine efficacies of 78, 91, and 96%, respectively. Interestingly, the study noted a significant overlap of antibody titers in the vaccinated individuals with break through infections vs. those without infection (9). The FDA and the CDC thus caution against using antibody levels to assess the level of protection against infection or disease in any individual patient.

However, in kidney transplant recipients not only is the seroconversion rate poor, but also the anti-S1/RBD Ig titer is low, suggestive of impaired virus neutralization in these patients as compared with others (10, 11).

### Measurement of cellular immunity after vaccination

Cellular immunity is an important arm of the immune system and can be measured by (SARS-CoV-2)-specific, interferon-γ (IFN-γ)-producing T and B cells, measured with an enzyme-linked immunospot (ELISpot) assay (12–14). Interferon gamma release assay (IGRA) is a measure of T cell response and is performed by isolating peripheral blood mononuclear cells (PBMCs) from subjects after vaccination. The PBMC's are thawed, counted, and stimulated with SARS-CoV-2 IGRA stimulation tube set. After stimulation, samples are centrifuged, and the supernatants used for subsequent quantitative assay using IFN-γ ELISA. In this test, the source of antigen specific IFN-γ production was mostly CD4 and sometimes CD8 T cells. The cellular immune response is traditionally not measured as the cutoffs for the protective CD4^+^ or cytolytic CD8^+^ T cell responses are not well-established. Viral-specific helper CD4^+^ T cell responses have been shown to correlate with quantitative antibody levels and neutralization titers. Unfortunately, assays to measure cellular response to COVID vaccination are not commercially available but have been used for research purposes.

## Response rates to COVID-19 vaccination in kidney and other solid organ transplant recipients

All vaccines available in the U.S. are designed to elicit an immune response against the spike protein. Response to immunization may be measured by clinical outcomes and by laboratory responses, assessing humoral and cellular immunity. Good response to COVID-19 vaccination is noted in healthy and even in dialysis subjects however the response in kidney transplant recipients is poor, ranging between 5 and 48% after 2 doses (15–17), with varying antibody titer response (11), as well as low percentages of neutralizing antibody inhibition as measured by surrogate viral neutralization test (SVNT).

Whether protection against COVID-19 after mRNA vaccination depends mainly on cellular (cytotoxic cells) or humoral (antibodies) adaptive immune effectors–termed mechanistic correlates of protection–is not yet entirely clear and might vary depending on factors such as the virus variant or patient characteristics. It is easy to speculate that antibodies may be essential to prevent infection (by blocking the RBD and preventing binding, fusion and cell entry of the virus) and that cellular immunity may be more important in controlling the disease. T-cell immunity after vaccination mimicked the poor humoral responses in some studies (10, 18), but was higher than humoral response in other (19) and had higher response after booster doses (20). In the study by Hall et al. (21) almost half of the patients who did not develop an antibody response, still had T-cell response. Their study found that after 2 doses of vaccination, anti-RBD antibodies were found in 34.5% of solid organ transplant recipients (SOTR's), however, a significant number of these patients did not show neutralization antibodies (28.5%).

Quantitative anti RBD levels seemed to correlate better with percent neutralizing antibodies. To date, there is no identified humoral or cellular correlate of immunity in SOTR's to predict protection from infection or severe COVID disease following either vaccination or infection.

For this reason, routine use of serology, testing the immune response to vaccination, is not recommended.

Furthermore, the immune response to vaccination is short-lived compared to the general population. In the study of SARS-CoV-2 antibody kinetics and durability, 73% of SOTRs had positive antibody titers 6 months following mRNA vaccination; titers increased in 27% over 6 months, decreased in 12%, and remained stable in 61% of patients over 6 months. This differs from healthy individuals, who had overall stability of antibody positivity over 6 months (11). In a study of 60 healthcare workers, divergent T and B-cell responses were noted following vaccination: antibody neutralization of Omicron was weak, but CD4 and CD8 responses against Omicron were maintained (22). This finding is consistent with the previously mentioned potentially different roles of antibodies and cellular immunity in preventing infection and controlling disease. In contrast to the positive effect against Omicron of the booster vaccination in healthy subjects (22), three doses of the mRNA-1273 vaccine resulted in very poor neutralizing activity against the Omicron BA1 variant in solid organ transplant recipients (the majority kidney and kidney-pancreas) (23).

### Factors associated with poor response to vaccination

Factors associated with poor response to vaccination include older age, diabetes, reduced GFR, higher mycophenolic acid dose, belatacept use, lower lymphocyte count, high-dose corticosteroids in the last 12 months, and treatment with B-cell depleting agents as Rituximab and antithymocyte globulins use within a year (17, 24–27). A multicenter, observational, case-control study including 132 consecutive kidney transplant recipients was done comparing 2 groups: Group A, with CNI/MMF/Pred (*n* = 104); Group B, with CNI/m-Tor-I/pred (*n* = 28) suggested that therapy with mTOR-I resulted in humoral and T cell–mediated immune response to COVID-19 vaccine (28). However, this result was not confirmed in a larger cohort of 1,037 subjects, which suggested the apparent benefit of mTOR-I regimen was a consequence of confounding by MMF (29). Use of immunosuppressants has been associated with the poor response to vaccination, but a French registry study that adjusted for comorbidities reported that the severity of COVID-19 in KTRs is related to their associated comorbidities, and not to chronic immunosuppression (30).

Studies focusing on clinical endpoints show that, even if 2 doses of vaccination result in low immune responses, there is still a significant decrease in mortality from COVID-19 in KTR from 12.6 to 7.7% in the UK registry study. Of note, this study included Pfizer (BNT162b2) and Oxford/ AstraZeneca (ChAdOx1-S) from their initial rollout in December 2020, until September 1, 2021, and hence precedes both Delta and Omicron variants of the virus (31).

Similarly, Aslam et al. (32) also found an 80% reduction in incidence of symptomatic COVID-19 infection after 2 doses of vaccination when compared to unvaccinated solid organ transplant recipients among 2,151 SOTRs at the University of California San Diego registry. This study was conducted prior to the emergence of Omicron variant.

Using a different design, Hall et al. conducted a multi-center propensity matched cohort study from 9 centers between March 2020 to September 2021 to ascertain whether there is a difference in outcome, once symptomatic COVID-19 develops, between unvaccinated (*n* = 220) and totally or partially (2 or 1 dose) vaccinated (*n* = 77) solid organ transplant recipients (33). They did not find a difference in the disease severity outcomes, duration of hospitalization, all cause and COVID-19 related mortality between the two groups. The result was the same when the fully vaccinated (2 doses) patients were considered as part of a sensitivity analysis. This study period was also before the emergence of Omicron variant.

## Multiple doses tend to increase antibody levels, but not in every patient

Response to vaccine varies from 5 to 48% after 2 doses (15–17), along with varying titer levels (11). In order to augment the antibody response, different strategies have been employed: multiple boosters, combination with heterologous vaccinations and decreasing immunosuppressant medications.

Three doses of mRNA vaccine are now recommended by CDC as the primary vaccination series in immunosuppressed patients followed by a fourth booster dose, to enhance protection against COVID-19 infection in SOTRs. For primary vector vaccine recipients, 2^nd^ dose with mRNA and additional booster also with mRNA vaccine is recommended.

There have been variable reports of response to vaccination after 3 doses, depending on the studied population. Good response was noted in some (20, 34, 35), while some continued to have poor response after the third dose (36).

Hall et al. reported an increase in neutralization antibody from 40 to 90% along with an increase in polyfunctional T-cells, following a third dose of mRNA vaccine (20). Booster doses have been associated with increased humoral response to wild type, however the neutralization effect was lower for Omicron variants (23). However, it does reduce severe COVID infection from Omicron, likely due to the effect of vaccination on the cellular immunity arm (37).

In a study by Karaba et al. 47 SOTRs were tested for anti-spike IgG antibody, pseudo neutralization activity and live virus neutralization against Variants of Concern (VOC), before and after third dose of SARS-CoV-2 vaccine and were compared to 15 healthy controls who received 2 doses of mRNA vaccine. Although third dose increased mean total anti spike IgG antibody by 1.64-fold, pseudo neutralization against VOC by 2.5-fold, and neutralizing antibodies by 1.4- fold against the delta variant, neutralizing antibody was still significantly lower than in healthy controls. 32% of SOT recipients had no detectable neutralizing antibody even after the third dose. Kidney transplantation was identified as a risk factor for decreased responsiveness to vaccine when compared to other SOT recipients, likely owing to higher immunosuppression. Mucosal and cellular immune responses were not evaluated in this study (36).

## Response to 3rd dose vaccine against variants of concern in SOT recipients

Kumar et al. (38) looked at neutralization response against VOC (alpha, beta and delta variants) following 3 rd dose mRNA vaccine compared to placebo in transplant recipients.

Sera was evaluated at 4–6 weeks following the 3 rd dose vaccination vs. placebo. The number of patients with improved virus neutralization response against all 3 variants–alpha, beta and delta –increased in recipients of 3 rd dose vaccine. The responders had antibodies against the wild type as well as other variants of concern, but at varying titers.

To look at the immune response against Omicron variant following 3 doses of vaccination in SOTRs, Kumar et al. (23) evaluated sera following 3 doses of mRNA vaccine at 1 and 3 months, after the third dose. At 1 month 18.3% (11/60) had detectable neutralizing antibody response to Omicron which further decreased at 3 months to 15.7% (8/51)-~5-fold reduction. The proportion of patients with positive antibody response to Omicron was lower than those against wild type and delta variants at both time points. Many patients with positive anti-RBD still had undetected Omicron specific neutralizing antibody, again highlighting poor immune response in SOTRs against emerging variants.

While some subjects continue to have poor response to third dose, subsequent boosters may further enhance the immune response (34).

## Response to 4th dose

Following 3 doses of vaccination, anti SARs CoV 2 antibodies were detected in about 60–70% of SOTRs (39). A case series in France (40) looked at the immune response to fourth dose mRNA vaccine in KTRs with 3 previous vaccinations. Of the 37 KTRs in the series, 5 (13.5%) had weak antibody response, while 31 (83.8%) had no response. Following 4rth dose with mRNA vaccine, 18 of 37 (48.6%) had detectable antibodies at 4 weeks. Among the 31 seronegative patients, 13 (41.9%) became seropositive but the mean antibody concentration remained low. Neutralizing antibodies at 4 weeks were not significantly different between the responders and non-responders to previous 3 doses, all had titers <64 IU/ml. Cellular response measured by IFN-γ was also low in both groups. In this study the neutralizing antibodies despite 4 doses of vaccination continued to remain low in both responders and non-responders, with no significant changes in their cellular immunity, although there was increased seroconversion.

Similarly, 4th dose vaccine did not increase neutralization against Omicron variant in a small observational cohort of 25 SOT recipients, although seropositivity, anti-Spike Ab levels and neutralizing activity against other variants had improved (41).

## Response to 5th dose

Eighteen SOTR were administered 5th dose vaccine in a case series (42). Fifteen of the 18 recipients received heterologous vaccine combinations. Two of 18 (11%) were seronegative prior to dose 5. Following dose 5, antibody titers were tested at 4 weeks. Seventeen of 18 had high antibody titers, while 1 patient continued to remain seronegative. The median anti RBD titer in those who had previously tested positive increased to 2,500 units/ml, with 16 of 17 (94%) ≥ 250 units/ml, and 12 of 17 ≥ 2,500 units/ml. This study demonstrated that in patients with weak vaccine induced antibody response, additional booster doses continue to enhance antibody levels. Many participants demonstrated antibody levels after dose 5 to levels observed in general population following 2 doses of mRNA vaccine series. However, despite 5 doses, 1 patient continued to remain seronegative (2 BNT, 2 Moderna and 1 Ad26.COV2. S) and was noted to be on high dose of CellCept at 2,500 mg/day. T-cell responses, neutralizing assay antibodies and memory B-cells were not assessed.

Due to poor serological response to Omicron variant with the available vaccines, Bivalent boosters has been approved and are now commercially available to provide additional protection against Omicron variant. In those who continue to have poor response to vaccination, reduction in immunosuppression is a consideration as described below (34).

## Heterologous vaccination strategy

There have been higher seroconversion rates induced by mRNA-1273 compared to BNT162b2 vaccine boosters, 49–26%, respectively in transplant recipients. This could be due to the higher dose with mRNA-1273 (3) and has led to trials of heterologous (mix and match) vaccinations for booster doses.

Several randomized control studies have evaluated immune response to heterologous vaccination against SARS-CoV-2 in the general population. Com-CoV (43) is a non-inferiority trial which showed heterologous vaccination schedule with adeno vector and mRNA vaccine was 76% efficacious against symptomatic and 100% efficacious against severe disease in the general population. Increasing the prime boost interval from 4 weeks to 8–12 weeks also showed continued protection against COVID infection in the general population (44). In these trials, highest anti spike antibodies and neutralizing antibodies were found in recipients with at least 1 mRNA containing regimen while increased T-cell response was noted in the vector virus recipients. COV-BOOST trial (45) looked at immunogenicity 3 months after the third dose boosters, and noted that the decay rate of humoral immune response was slower with vector virus booster vaccine (Ad26.COV2.S) when compared to mRNA vaccines, while the decay of cellular responses was similar in all vaccine schedules.

In CVIM 4 study, a RCT of 85 KTRs, recipients who had been previously received primary series consisting of either two doses of CoronaVac or followed by one dose of ChAdOx1nCoV-19 vaccine or two doses of ChAdOx1 nCoV-19 vaccine were randomized to receive either an mRNA (M group; *n* = 43) or viral vector (V group; *n* = 42, ChAdOx1nCoV-19) booster. At 2 weeks post-additional dose, there was no difference in the measured B and T cell immune responses between the two groups (46).

Heinzel et al. (47) looked at seroconversion at 3 months following 3 rd dose heterologous vs. homologous vaccine in KTRs. Their initial RCT trial looking at seroconversion 4 weeks after heterologous (vector) vs. homologous (mRNA) 3 rd dose vaccination in 201 KTRs, showed no significant difference in the development of SARS COV 2 spike protein antibodies.

But at 3 month follow up, although seroconversion was similar between the 2 groups, significantly larger number of recipients in the vector group reached antibody levels >141 and >264 BAU/ml. Also, antibody levels in the seroconverted patients further increased from 1 to month 3 in the vector group, while they remained unchanged in the mRNA group (Median increase: mRNA = 1.35 U/ml and vector = 27.6 U/ml, *p* = 0.004). The group reported that despite a similar overall seroconversion rate at 3 months following third dose vaccination in kidney transplant recipients, a heterologous 3rd booster vaccination with Ad26.COV2.S resulted in significantly higher antibody levels in responders. This study also looked at the T cell response at 1 month f/u in 18 patients among the top responders to the 3^rd^ dose from both groups to see if higher T-cell response led to the subsequent increase in antibody levels in the vector group but found SARS-CoV-2-specific CD4 and CD8 T-cells comparable between the 2 groups.

## Vaccination with decreased immunosuppressants

Immunosuppressant use is associated with decreased response to vaccination. There are on ongoing randomized controlled trials to further evaluate this association (48).

In the small study of 29 KTRs who had not responded after 3 vaccination doses, the authors held mycophenolic acid (MPA) for 5 weeks during the 4^th^ booster. They observed seroconversion in 76% of patients. The overall proportions of spike reactive CD4^+^ T cells remained unaltered after the fourth dose; frequencies were positively correlated with specific IgG levels. Importantly, antigen-specific proliferating Ki67^+^ and *in vivo* activated PD1^+^ T cells significantly increased after re-vaccination during MPA hold, whereas cytokine production and memory differentiation remained unaffected (35).

In a larger study (34), 4,277 vaccinations against SARS-CoV-2 in 1,478 patients were analyzed.

Serological response was 19.5% after 2 doses, and increased to 29.4, 55.6, and 57.5% after third, fourth, and fifth vaccinations, resulting in a cumulative response rate of 88.7%. In patients with calcineurin inhibitor and MPA maintenance immunosuppression, pausing MPA and adding 5 mg prednisolone equivalent before the fourth vaccination increased the serological response rate to 75% in comparison to no dose adjustment (52%) or dose reduction (46%). Belatacept-treated patients continued to have a poor response rate of 8.7% (4/46) after three vaccinations and 12.5% (3/25) after four vaccinations (34).

## Pre and post exposure prophylaxis

Tixagevimab and cilgavimab (tradename Evusheld) are neutralizing monoclonal antibodies directed against different epitopes of the receptor-binding domain of SARS-CoV-2 spike protein that have been associated with a lower risk of SARS-CoV-2 infection when used for preexposure prophylaxis. It contains 2 monoclonal antibodies that bind to non-overlapping epitopes of the spike receptor binding protein and neutralize the virus (49, 50). It is administered intramuscularly, has a long half-life and is dosed every 6 months. In the PREVENT trial, symptomatic COVID-19 occurred in 8 of 3,441 participants (0.2%) in the AZD7442.

(Evusheld) group, and in 17 of 1,731 participants (1.0%) in the placebo group. Extended follow-up at a median of 6 months showed a relative risk reduction of 82.8% (95% CI, 65.8–91.4). Side effects in PREVENT were headache (6%) and fatigue (4%). There were some serious cardiac events e.g., myocardial infarction, cardiac failure, arrhythmia in the antibody group than in the placebo group (0.6 vs. 0.2%) (51). It is fully active against the Delta variant of SARS-CoV-2. The Omicron variant was not prevalent during clinical trials of Evusheld (51).

In a retrospective study of 222 KTRs during the COVID Omicron variant, (52) breakthrough SARS-CoV-2 infections occurred in 11 (5%) of SOTRs who received tixagevimab/cilgavimab and in 32 (14%) of SOTRs in the control group (*p* < 0.001). In the tixagevimab/cilgavimab group, SOTRs who received the 150–150 mg dose had a higher incidence of breakthrough infections compared to those who received the 300–300 mg dose (*p* = 0.025). There were only mild side effects.

However, efficacy of the available antibodies varies depending on the variant (53). The authors tested the efficacy of antibody against various COVID-19 variants and reported the tixagevimab–cilgavimab combination inhibited beta, gamma, and omicron; however, the Focus reduction neutralization test (FRNT_50_) values of this combination were higher by a factor of 24.8–142.9 for omicron than for beta or gamma, respectively, suggesting that a much higher concentration was needed for neutralizing the omicron variant.

In the transplant community, depending on each center's protocol, Evusheld is administered every 6 months to at risk patients. The at-risk group is defined differently by each center. Common practice at larger centers is to give the antibody 1 week after transplant. Some centers also administer to those who did not have an antibody response to vaccinations, those who received rituximab or recent treatment for rejection, or those within 1 year of transplant.

Unfortunately, new VOC showing extensive escape to tixagevimab–cilgavimab (Omicron BA.4.6, BA.4.7, and BA.5.9) have already been detected, so it is unclear how effective this strategy will be in the near future (54).

KTRs who have reduced response to vaccinations even with repeated doses, are at high risk of severe COVID infections requiring hospitalizations and mechanical ventilations. Previously, convalescent plasma infusion was undertaken to provide passive immunity. There are different monoclonal anti-spike antibody formulations available for post exposure prophylaxis and are used depending on the type of COVID 19 variants (55). They can be used in combination with anti-viral medications: remdesivir, molnupiravir or nirmatrelvir/ritonavir, reducing COVID-19-related morbidity and mortality in SOTR (56, 57). However, the monoclonal antibodies have different efficacy against different variants (53).

The new therapies that are being investigated include ALVR109. ALVR109, is an investigational, off-the-shelf T cell therapy, consisting of partially HLA-matched, polyclonal (CD4+ and CD8+) SARS-CoV-2–specific T cells expanded from the peripheral blood of convalescent healthy donors seropositive for SARS-CoV-2. *In vitro*, ALVR109 has shown the ability to target clinically important variants of SARS-CoV-2, including Alpha, Beta, Gamma, Delta, Epsilon, and Kappa (58).

## Other immunosuppression considerations

Anti-viral medications molnupiravir, nirmatrelvir/ritonavir reduces COVID-19-related morbidity and mortality in SOTR (56, 57) and has similar efficacy as bebtelovimab used for post exposure prophylaxis. In a retrospective cohort study of 3,607 high-risk patients, bebtelovimab was compared with nirmatrelvir-ritonavir for treatment of COVID-19 among older patients, immunosuppressed patients, and those with multiple comorbidities. The rates of progression to severe disease after bebtelovimab (1.4%; 95% confidence interval: 1.2, 1.7) was not significantly different from nirmatrelvir-ritonavir treatment (1.2%; 95% confidence interval: 0.8, 1.5) (59).

The use of Nirmatrelvir/ritonavir in transplant recipients is complicated by the strong interaction with CNI and mTOR-i, which need to be discontinued during this therapy, and requires close monitoring of levels after reinitiating.

Maintenance immunosuppression should also be decreased depending on the severity of COVID-19. Decreased immunosuppression is generally not associated with increased rejection. In a study of 64 KTRs with COVID-19, 31 with acute COVID-19 (<4 weeks from diagnosis) and 33 with post-acute COVID-19 (>4 weeks postdiagnosis) blood transcriptomes were examined (60). The authors reported upregulation of neutrophil and innate immune pathways but downregulation of T cell and adaptive immune activation pathways with acute infection. This finding was independent of lymphocyte count, despite reduced immunosuppressant use, indicating that blood lymphocytes are not the primary source. Hence, the decreased adaptive immunity could be protective. Furthermore, serum inflammatory cytokines followed an opposite trend (*i.e*., increased with disease severity), also indicating that blood lymphocytes are not the primary source and suggested role of cytokine inhibitors during the COVID infection.

## Conclusion

Kidney transplant recipients mount lower humoral and cellular response to COVID vaccinations when compared to the general population. Although multiple booster doses increase anti spike antibody titers in this group, they continue to remain much lower compared to the general population, and do not correlate well with neutralization against the Omicron variant. There is no defined cutoff value for humoral or cellular immunity that is identified to provide immunity against COVID infection or prevent breakthrough infections. There is growing evidence to show that cellular immunity is much more preserved in kidney and other solid organ transplant recipients when compared to humoral immunity over a period of 6 months and could play a role in preventing severe COVID infection. Recently approved bivalent COVID booster vaccination provides better protection against Omicron variant and should be recommended to all SOTRs. Pre exposure prophylaxis with Evusheld, especially in early posttransplant period, or following treatment for rejection, can help prevent COVID infection. Reduction in immunosuppression can be considered to improve vaccine response in low immunogenic groups. Post exposure monoclonal antibodies and antiviral medication therapy have shown good response in kidney transplant recipients.

## Author contributions

All authors listed have made a substantial, direct, and intellectual contribution to the work and approved it for publication.

## Conflict of interest

The authors declare that the research was conducted in the absence of any commercial or financial relationships that could be construed as a potential conflict of interest.

## Publisher's note

All claims expressed in this article are solely those of the authors and do not necessarily represent those of their affiliated organizations, or those of the publisher, the editors and the reviewers. Any product that may be evaluated in this article, or claim that may be made by its manufacturer, is not guaranteed or endorsed by the publisher.
